# A Comparative Analysis of Doherty and Outphasing MMIC GaN Power Amplifiers for 5G Applications

**DOI:** 10.3390/mi14061205

**Published:** 2023-06-07

**Authors:** Victoria Díez-Acereda, Sunil Lalchand Khemchandani, Javier del Pino, Ayoze Diaz-Carballo

**Affiliations:** Institute for Applied Microelectronics (IUMA), Universidad de Las Palmas de Gran Canaria (ULPGC), 35017 Las Palmas de Gran Canaria, Spain; sunil@iuma.ulpgc.es (S.L.K.); jpino@iuma.ulpgc.es (J.d.P.); ayoze.diaz104@alu.ulpgc.es (A.D.-C.)

**Keywords:** Doherty PA (DPA), outphasing PA (OPA), power added efficiency (PAE), output power back-off (OBO), GaN, fifth generation (5G)

## Abstract

A comparison between a fully integrated Doherty power amplifier (DPA) and outphasing power amplifier (OPA) for fifth Generation (5G) wireless communications is presented in this paper. Both amplifiers are integrated using pHEMT transistors from the OMMIC’s 100 nm GaN-on-Si technology (D01GH). After a theoretical analysis, the design and layout of both circuits are presented. The DPA uses an asymmetric configuration where the main amplifier is biased in class AB and the auxiliary amplifier is biased in class C, while the OPA uses two amplifiers biased in class B. In the comparative analysis, it has been observed that the OPA presents a better performance in terms of maximum power added efficiency (PAE), while the DPA provides higher linearity and efficiency at 7.5 dB output back-off (OBO). At a 1 dB compression point, the OPA exhibits an output power of 33 dBm with a maximum PAE of 58.3% compared to 44.2% for the DPA for an output power of 35 dBm, and at 7.5 dB OBO, the DPA achieves a PAE of 38.5%, while the OPA achieves 26.1%. The area has been optimized using absorbing adjacent component techniques, resulting in an area of 3.26 mm^2^ for the DPA and 3.18 mm^2^ for the OPA.

## 1. Introduction

The fifth generation (5G) allows 1000 times more data transmission capacity than the 4G technology, 10 to 100 times more connectivity between devices, 5 times more responsiveness, 90% energy savings and the same efficiency everywhere. In other words, the goal of 5G is to increase usage, speed and services in telecommunication, as well as reducing energy consumption. However, there is a hidden threat behind the promise of 5G. This new wireless communication can deliver much more data than current networks, but at a higher energy cost. Fears about energy efficiency are beginning to surface and, therefore, the use of new alternative energy methods has become an important area of research. The International Telecommunication Union (ITU) and the Third Generation Partnership Project (3GPP) have published challenging measurable requirements on the data rates, latency and reliability that a network needs to support 5G and distinguish itself from 4G [1,2]. The key performance indicators (KPIs) for 5G wireless technology are grouped into three main categories: the millimetre-Wave (mm-Wave), ultra-reliable low latency communication (URLLC) and the massive machine-type communication (mMTC). mm-Wave new radio (NR) focuses on enhancing mobile broadband (eMBB) to increase data bandwidth and efficient connectivity. This enables better upload and download speeds compared to 4G/LTE networks, and it uses a different set of radio frequency bands. On the other hand, ultra-reliable low latency communication (URLLC) focuses on achieving low latency for mission-critical applications, such as factory automation, remote robotic surgery and self-driving cars. URLLC requirements are still being standardized and are part of the different 3GPP releases [3]. Lastly, massive machine-type communication (mMTC) focuses on low-power, high-density targeting of narrowband Internet of Things (NB-IoT) applications and smart devices [4]. The frequency bands for 5G mobile networks are organized into two different frequency ranges (FR): FR1 and FR2. FR1 ranges from 450 MHz to 6 GHz. These bands are also known as Sub-6 GHz. FR2 ranges from 24.25 GHz to 52.6 GHz and the bands are named mm-Wave [5,6].

The demand of wide bandwidth and high peak-to-average power ratio (PAPR) have been increasing in modern wireless communication systems such as 5G because they use complex-modulated signals. This means that the transmission chains, especially the power amplifiers (PA) must work with a higher back-off output power (OBO), providing users with a high efficiency, as well as maintaining the linearity. Several PA architectures have been developed to improve efficiency at back-off levels [7,8,9,10,11]: envelope-tracking PA, envelope elimination and restoration PA, Doherty PA (DPA) and outphasing PA (OPA).

The envelope tracking (ET) [12,13] and envelope elimination and restoration (ERR) [14,15] techniques are based on the bias modulation principle, in which the RF power’s collector/drain supply is varied with the output envelope, thus resulting in higher efficiency and linearity of the transistor over a broad range of output power. However, the drawback of these PAs is the limited modulation bandwidth of the supply modulator as its performance depends upon the efficiency of the power amplifier, peak power and dynamic range.

The other techniques are based on the principle of active load modulation, which are the Doherty [16] and Outphasing [17] techniques. However, one of the main problems with these architectures is the high occupied area due to the use of power dividers and combiners at the input and output. For very high frequencies, these elements are usually implemented with transmission lines, while for lower frequencies, such as those used in Sub-6 GHz 5G, they are implemented with lumped elements. A design methodology presented in [18] involves absorbing adjacent components into one single element for an outphasing amplifier, which reduces the complexity of the output network and decreases the occupied area.

The objective of this paper is to compare the conventional DPA architecture with the OPA at 3.6 GHz. Both circuits will be implemented with lumped components, and the technique presented in [18] will be utilized to reduce their area. The design of both amplifiers will use a pHEMT GaN-on-Si transistor with depletion mode from the OMMIC foundry. Section 2 and Section 3 describe the operating principles of the Doherty and outphasing PAs, respectively, and also present the basic circuit configuration of both PAs. Section 4 shows the comparative simulation results of the two PAs, followed by conclusions in Section 5.

## 2. Doherty Amplifier

### 2.1. Doherty Amplifier Analysis

**Figure 1:**
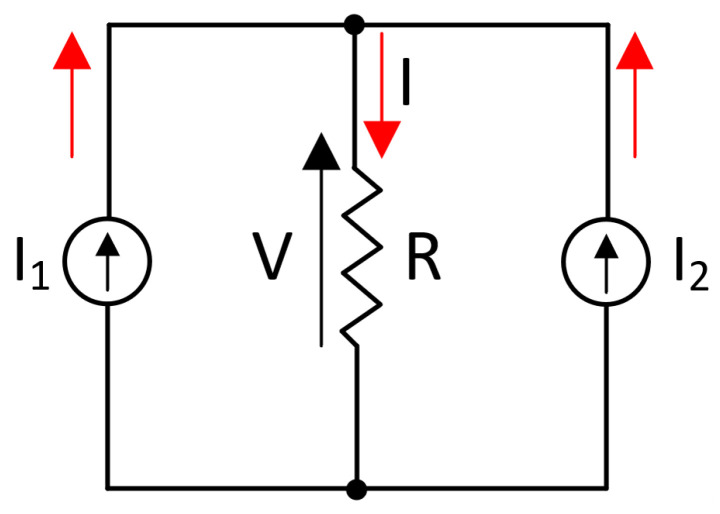
Active load-pull technique.

The Doherty PA was first proposed by W. H. Doherty in 1936 with the use of vacuum tubes. This new device was first used in a 50 kW transmitter radio station in Kentucky, improving the efficiency of the RF power amplifier. Nowadays, with the release of new communication standards, the amplifier has been reinvented by mobile communication systems using semiconductor devices at higher frequencies. The Doherty amplifier is based on the active load-pull technique, which has been widely explained in the literature [7,16,19]. However, to understand its operation, this explanation will be made, so the following scheme of the Doherty amplifier must be considered in Figure 1, where two current sources are connected to an impedance R [7,19].

Applying Kirchhoff and Ohm’s laws in the circuit, the voltage across the impedance is given by [7]:

(1) $$V=I\cdot R=(I_{1}+I_{2})\cdot R$$

where $I_{1}$ and $I_{2}$ are the currents supplied by the sources $I_{1}$ and $I_{2}$, respectively. Therefore, if the currents are not null, the load impedance seen by the source $I_{1}$ is [7]:

(2) $$R_{1}=\frac{V}{I_{1}}=R\cdot \frac{\left(I_{1}+I_{2}\right)}{I_{1}}=R\cdot \left(1+\frac{I_{2}}{I_{1}}\right)$$

Similarly, the load resistance seen by the current source $I_{2}$ is given by [7]:

(3) $$R_{2}=\frac{V}{I_{2}}=R\cdot \frac{\left(I_{1}+I_{2}\right)}{I_{2}}=R\cdot \left(1+\frac{I_{1}}{I_{2}}\right)$$

**Figure 2:**
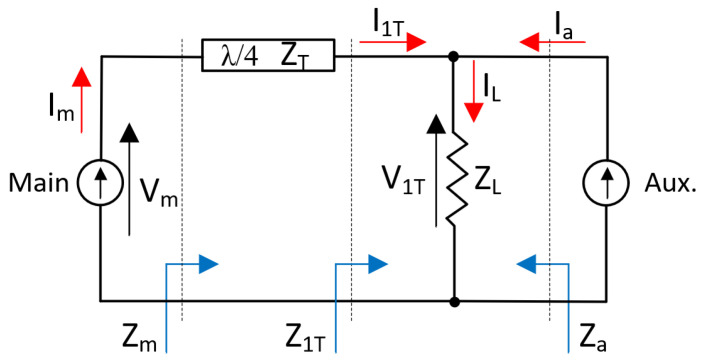
Doherty load modulation principle.

From Equations (2) and (3), we can observe that the impedances ($R_{1}$ and $R_{2}$) seen by one current source are controlled by the current level of the other one. Therefore, changing the impedance level seen by one current source results in a variation of the voltage across the impedance R. Now, consider that the ideal current sources are active devices such as transistors (main and auxiliary). To maximize the efficiency of a device (main), while its output load is changing (by the current supplied by the auxiliary device), the voltage swing across it must be maintained constant. To guarantee this condition, a λ/4 transmission line is added between the load and the main source, as shown in Figure 2.

Now, the impedance seen at each side of the load resistors can be expressed as shown in (4) and (5) [7].

(4) $$Z_{1T}=Z_{L}\cdot \left(1+\frac{I_{a}}{I_{1T}}\right)$$

(5) $$Z_{a}=Z_{L}\cdot \left(1+\frac{I_{1T}}{I_{a}}\right)$$

The λ/4 transmission line (T-Line) after the main amplifier acts as an impedance transformer. The relationship between the voltages and currents placed at the sides of this T-Line can be written as follows [7]:

(6) $$V_{1T}\cdot I_{1T}=V_{m}\cdot I_{m}$$

(7) $$Z_{T}^{2}=Z_{m}\cdot Z_{1T}$$

Rearranging (6) and (7), $I_{1T}$ and $V_{1T}$ can be expressed as [7]:

(8) $$I_{1T}=\frac{V_{m}}{Z_{T}}$$

(9) $$V_{1T}=Z_{T}\cdot I_{m}$$

Substituting (8) and (9) in (4) and (5) and rearranging the equations, $Z_{1T}$ and $Z_{a}$ can be rewritten as [7]:

(10) $$Z_{1T}=Z_{L}\cdot \left(1+\frac{I_{a}\cdot Z_{1T}}{V_{m}}\right)$$

(11) $$Z_{a}=Z_{L}\cdot \left(1+\frac{V_{m}}{Z_{T}}\cdot I_{a}\right)$$

Thus, clearing $Z_{m}$ from (8) and substituting $Z_{1T}$, the output impedance seen by the main amplifier is [7]:

(12) $$Z_{m}=\frac{Z_{T}^{2}}{Z_{1T}}=\frac{Z_{T}^{2}}{Z_{L}\cdot \left(1+\frac{I_{a}\cdot Z_{1T}}{V_{m}}\right)}$$

Moreover, the effective load impedances seen by the current sources ($Z_{m}$ and $Z_{a}$) can be expressed as a function of $Z_{T}$ and $Z_{L}$ as it follows [20]:

(13) $$Z_{a}=\frac{V_{a}}{I_{a}}=\frac{Z_{T}\cdot I_{m}}{I_{a}}$$

Noting that $V_{m}$ = $I_{m}$·$Z_{m}$, by addition and subtraction, we can have [20]:

(14) $$Z_{m}=\frac{Z_{T}^{2}}{Z_{L}}\cdot \left(1-\frac{Z_{L}}{Z_{T}}\cdot \frac{I_{a}}{I_{m}}\right)$$

**Figure 3:**
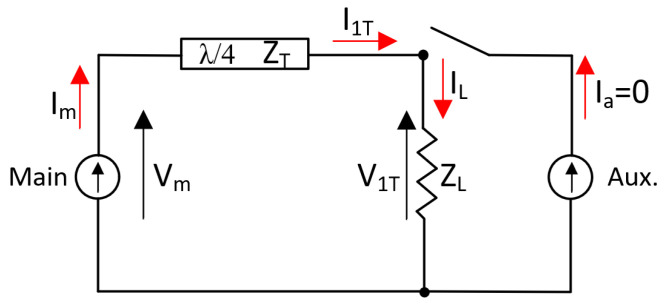
Doherty operation when the main amplifier is ON and auxiliary amplifier is OFF.

Typically, standardized characteristic impedances for the quarter-wavelength line is $Z_{T}$ = $Z_{0}$, while the load is $Z_{L}$ = $Z_{0}/2$. Assuming that there are identical currents at saturation, the effective impedances at different power levels can be easily calculated using (13) and (14). Therefore, when the input signal is low, only the main amplifier is active, while the auxiliary amplifier remains inactive due to its Class-C biasing. This is because the auxiliary amplifier requires a higher input signal to initiate current flow. This is called the low-power region. Figure 3 shows the operating scheme of the DPA when the main device is on and the auxiliary one is off, and the impedances are given by [20]:

(15) $$Z_{a}=\infty$$

(16) $$Z_{m}=\frac{Z_{T}^{2}}{Z_{L}}=2\cdot Z_{0}$$

Conversely, when the input signal is high, the main amplifier reaches saturation, resulting in the first efficiency peak. Under these conditions, both amplifiers operate simultaneously and enter the so-called Doherty region. In this region, the second efficiency peak is achieved at maximum output power. Figure 2 shows the operating scheme of the DPA when both amplifiers are on, and the impedances are given by [20]:

(17) $$Z_{a}=Z_{0}$$

(18) $$Z_{m}=\frac{Z_{T}^{2}}{Z_{L}}\cdot \left(1-\frac{Z_{L}^{2}}{Z_{T}}\right)=Z_{0}$$

**Figure 4:**
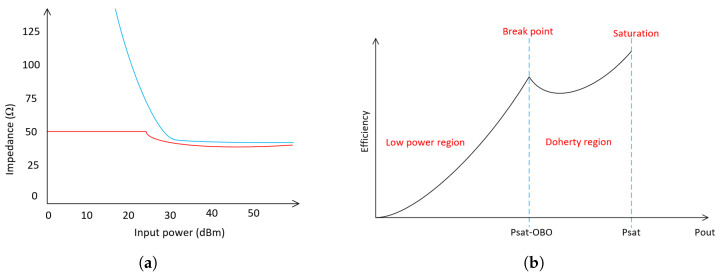
Doherty theoretical behavior. (**a**) Load trajectories: main amplifier (red) and auxiliary amplifier (blue). (**b**) Efficiency curves of the DPA.

To conclude, the load of the main amplifier goes from 2$Z_{0}$ to $Z_{0}$, while the auxiliary amplifier is modulated from *∞* to $Z_{0}$, as it is represented in Figure 4a. The theoretical expected efficiency behavior is reported in Figure 4b.

**Figure 5:**
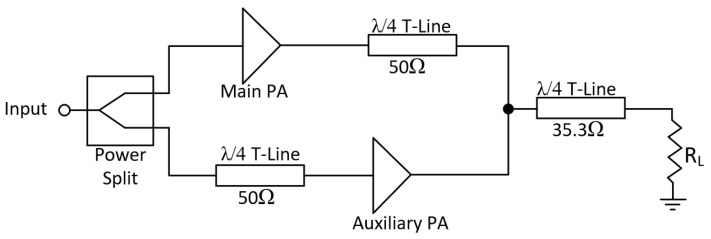
Doherty amplifier blocks diagram.

Therefore, DPAs are commonly implemented according to the configuration depicted in Figure 5. This setup comprises a power splitter, a main (or carrier) amplifier, and an auxiliary (or peaking) amplifier. As shown in the figure, the main PA is followed by a λ/4 T-Line, which is utilized for accurate load modulation. To ensure delay matching, another λ/4 T-Line is inserted at the input of the auxiliary amplifier [16].

### 2.2. Design and Implementation

The design of the DPA is described in this subsection. As recommended by the foundry, for the design of power amplifiers, a 100 nm GaN-on-Si transistor was used to design the main and auxiliary amplifiers. These transistors have been modeled for use in both small and large signal simulation. An asymmetric configuration is adopted where the peaking amplifier is larger than the main one. The main amplifier is biased in Class-AB and the auxiliary in Class-C, so that the gate voltage (VGS) and drain voltage (VDS) in the main amplifier are −1.5 V and 9 V, respectively, while in the auxiliary amplifier, the VGS is −3.1 V and the VDS is 12 V.

**Figure 6:**
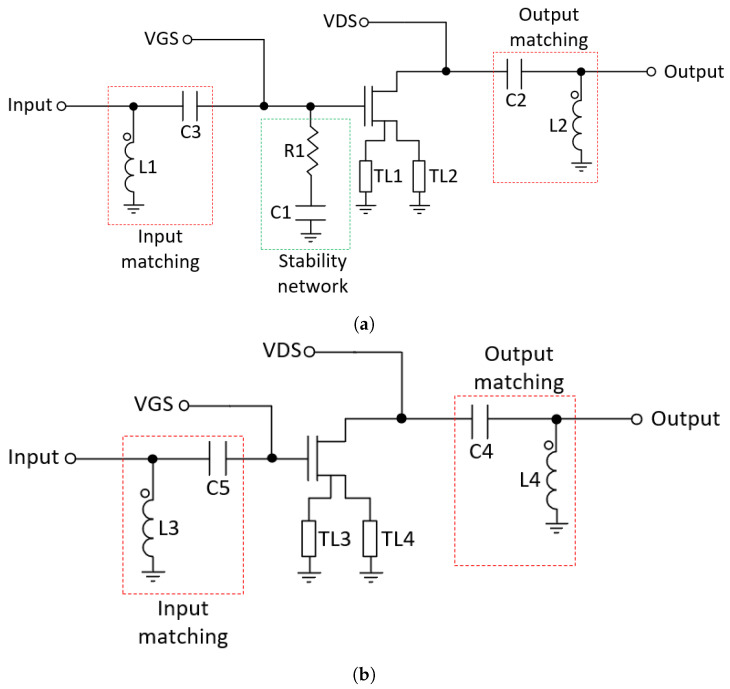
Doherty’s amplifiers. (**a**) Main amplifier. (**b**) Auxiliary amplifier.

Stability is a crucial part of a PA design, which requires special precautions to avoid unwanted oscillations. In order to avoid such instabilities, an RC network is added in the main amplifier (R1 and C1) [7,21] to make the amplifier unconditionally stable, as seen in Figure 6a. The inductors L1 and L2 and capacitors C3 and C2 are used to match the amplifier for maximum PAE. On the other hand, as shown in Figure 6b, the auxiliary amplifier does not need a stabilization network since it is stable. Inductors L3 and L4 and capacitors C4 and C5 make up the input and output matching networks of the auxiliary amplifier. The transmission lines (TL1-TL4) are used to connect the source of the transistors to ground.

A hybrid coupler was chosen to split the input power between the main and auxiliary amplifiers. This type of hybrid provides a 90-degree phase shift between its outputs [21], so the λ/4 T-line at the input of the auxiliary amplifier can be eliminated, reducing the circuit area.

**Figure 7:**
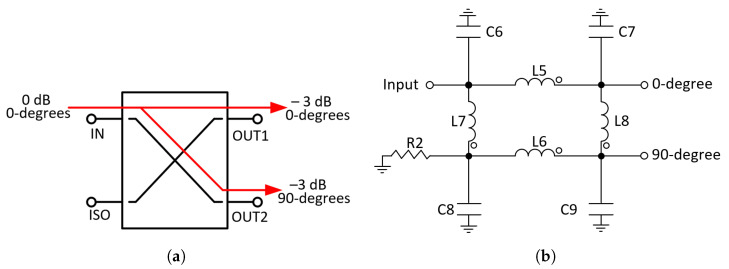
Power divider schematic. (**a**) Block diagram hybrid coupler. (**b**) Hybrid coupler with lumped elements.

The design of this component is relatively straightforward. The basic configuration is shown in Figure 7a. When power is introduced to the input port, half the power (−3 dB) flows to the 0-degree port (output 1), and the other half is coupled to the 90-degree port (output 2). The reflections from the mismatches are sent back to the output ports and they either flow directly to the isolated port or cancel at the input. This configuration ensures a high degree of isolation between the two output ports and the two input ports without any unwanted interaction between them [22].

**Figure 8:**
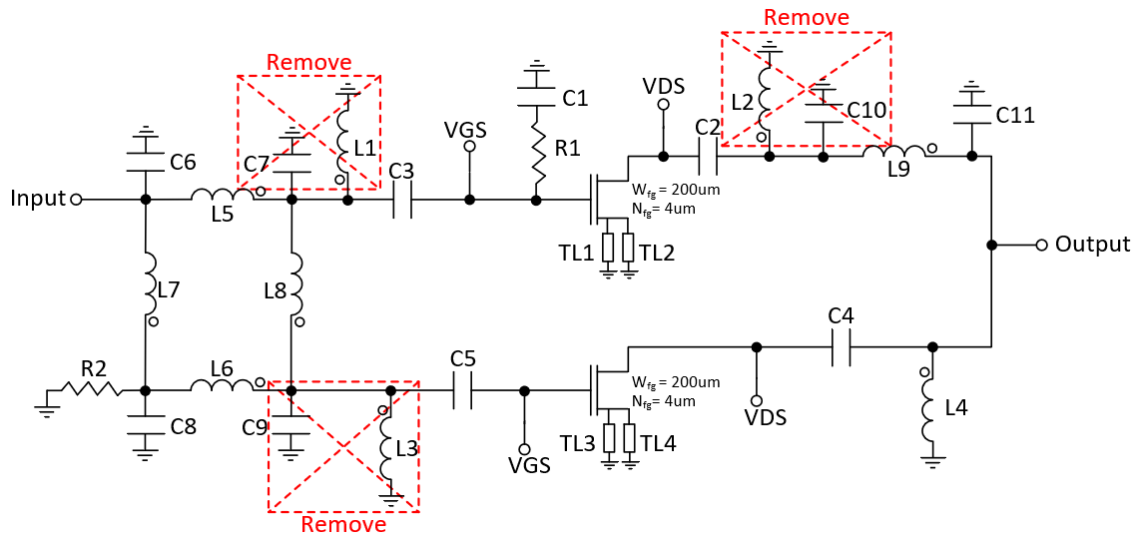
Doherty schematic.

In the first approach, the output network and the input hybrid of the DPA were designed with transmission lines, but due to its large area (7700 μm × 81 μm), they were discarded. Therefore, the output λ/4 T-line was replaced by a lumped-elements T-line using a π-network. This network is composed of capacitors (C10 and C11) and inductor (L9) [23]. Likewise, the input hybrid was implemented with lumped elements using inductors (L5, L6, L7 and L8), capacitors (C6, C7, C8 and C9) and resistor (R2) at the isolated port (see Figure 7b). The resulting schematic of the DPA is shown in Figure 8.

**Figure 9:**
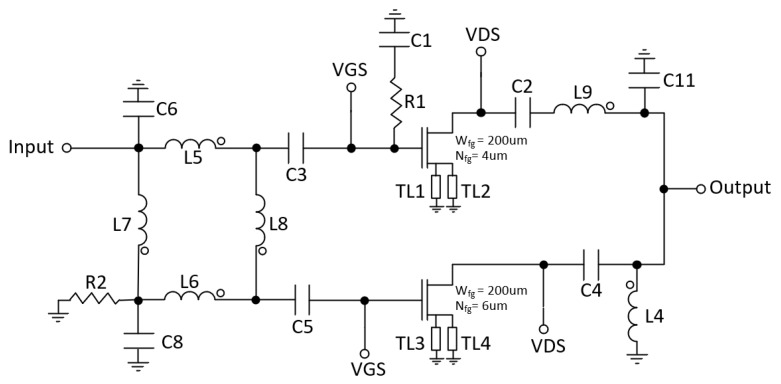
Final Doherty schematic.

As shown in Figure 8, there are parallel inductors with capacitors that can be eliminated by resizing other passive components. However, it is important to note that these simplifications cannot be made simultaneously. Instead, they have been implemented sequentially, optimizing the PAE after each individual change. Figure 9 and Table 1 illustrate the simplified schematic and the corresponding component values, respectively. In the schematic, Wfg and Nfg represent the width and number of gate fingers of the transistors, while W and L denote the width and length of the T-lines.

**Figure 10:**
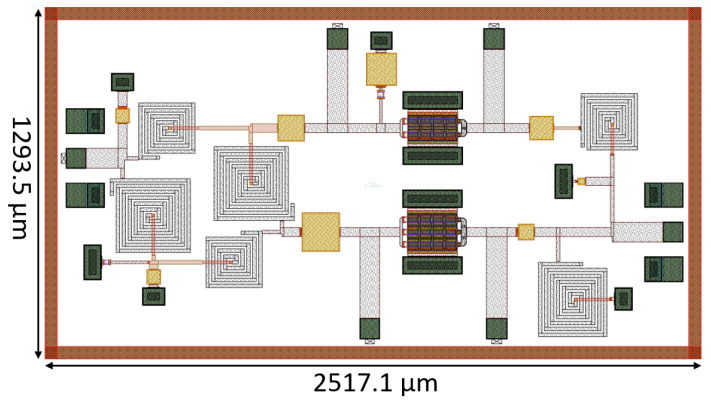
Doherty layout.

The layout of the DPA described in this paper was implemented using the 100 nm GaN-on-Si OMMIC process (D01GH). Figure 10 illustrates the final layout, which occupies an area of 2517.1 μm × 1293.5 μm (including PADs). The DC biasing PADs are located at the top and bottom of the chip, while Ground–Signal–Ground (GSG) RF input and output PADs are placed on the left and right sides. RF bias choke inductors were not included due to their high value.

## 3. Outphasing Amplifier

### 3.1. Outphasing Amplifier Analysis

Although this analysis can be found in the literature [7,17,24], in this subsection, the Outphasing amplifier is analyzed to provide a theoretical basis that helps to understand the results obtained. In 1935, Henri Chireix introduced the term outphasing for the first time in his paper “High Power Outphasing Modulation” [17]. This architecture emerged to reduce power consumption in tube-based transmission stations. Several years later, its application was expanded to microwave frequencies, and it became known as linear amplification using nonlinear components (LINC), which shows outphasing as a technique that produces a linear modulation by combining the output of non-linear amplifiers. If the two PA outputs are in opposite phases, output power is increased. Therefore, the combiner can be treated as a vector summation element.

**Figure 11:**
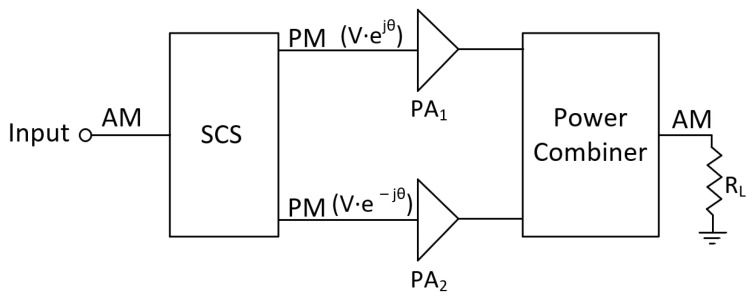
Outphasing amplifier blocks diagram.

Figure 11 illustrates a block diagram of the outphasing power amplifier, in which the input signal component separator (SCS) network converts the amplitude-modulated (AM) signal into two phase-modulated (PM) signals with opposite phases and constant envelopes. These two signals are then combined to generate an output AM signal after being amplified by two highly efficient amplifiers.

**Figure 12:**
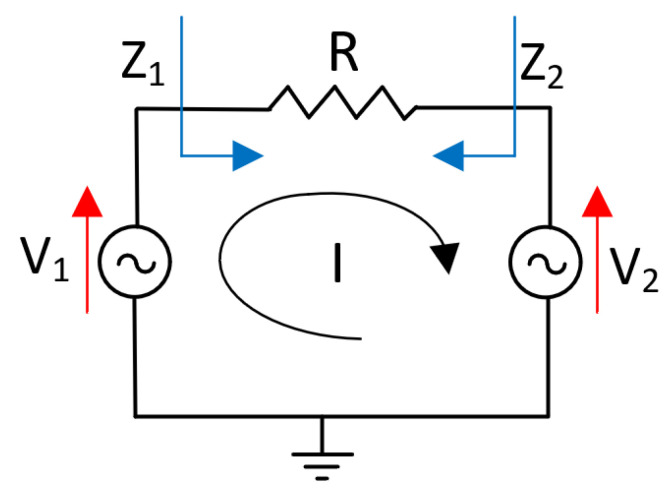
Outphasing load modulation principle.

To understand the operation principle of the outphasing PAs, a simplified concept of the load modulation technique is represented in Figure 12. The PAs are assumed to act as ideal voltage sources with equal amplitudes and opposite phases and are connected to a common series load resistance [7].

The voltage sources can be described by [7]:

(19) $$V_{1}=V\cdot e^{j\theta }$$

(20) $$V_{2}=V\cdot e^{-j\theta }$$

Applying the second Kirchhoff’s law in Figure 12, the circulating current can be written as [7]:

(21) $$I=\frac{V}{R}=\frac{\left(V_{1}-V_{2}\right)}{R}$$

The effective load seen by the current sources $Z_{1}$ and $Z_{2}$ is [7]:

(22) $$Z_{1}=\frac{V_{1}}{\left(V_{1}-V_{2}\right)}\cdot R=\frac{R}{2}\cdot \frac{\left(cos\cdot \theta +j\cdot sin\cdot \theta \right)}{j\cdot sin\cdot \theta }=\frac{R}{2}\cdot \left(1-j\cdot ctg\cdot \theta \right)$$

(23) $$Z_{2}=\frac{V_{2}}{\left(V_{1}-V_{2}\right)}\cdot R=\frac{R}{2}\cdot \frac{\left(cos\cdot \theta -j\cdot sin\cdot \theta \right)}{j\cdot sin\cdot \theta }=\frac{R}{2}\cdot \left(1+j\cdot ctg\cdot \theta \right)$$

From Equations (22) and (23), it is clear that each PA varies the load seen by the other according to the value of the outphasing angle θ.

**Figure 13:**
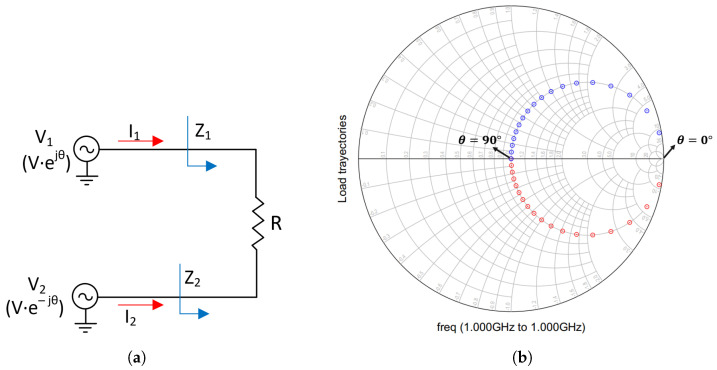
Load trajectories of the Outphasing PA for a phase sweep from 0-degree to 90-degree without compensation. (**a**) Schematic. (**b**) Simulation results. $Z_{1}$: blue trajectory and $Z_{2}$: red trajectory.

To verify the above equations, the schematic in Figure 13a was simulated. As seen in Figure 13b, when θ is equal to 0-degree, the output power is null, and as a result, the load impedances act as an open circuit. However, when θ is 90-degrees, the load impedances are purely resistive (R/2) and the output power is maximum. For the rest of the values, the impedances present a substantial imaginary part, and therefore the efficiency decreases.

**Figure 14:**
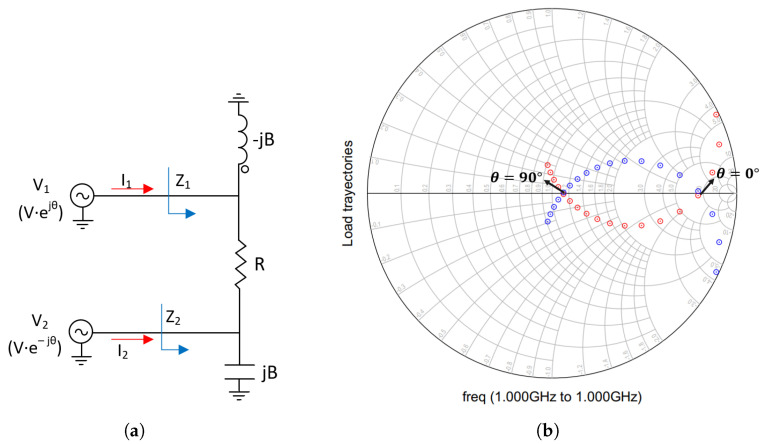
Load trajectories of the Outphasing PA for a phase sweep from 0-degree to 90-degree with compensation. (**a**) Schematic. (**b**) Simulation results. $Z_{1}$: blue trajectory and $Z_{2}$: red trajectory.

However, by adding two shunt-compensating reactive elements (see Figure 14a), a second efficiency peak can be obtained. These shunt-compensating reactive elements have equal but opposite susceptance. As shown in Figure 14b, the load trajectories of the two branches cross the real axis.

**Figure 15:**
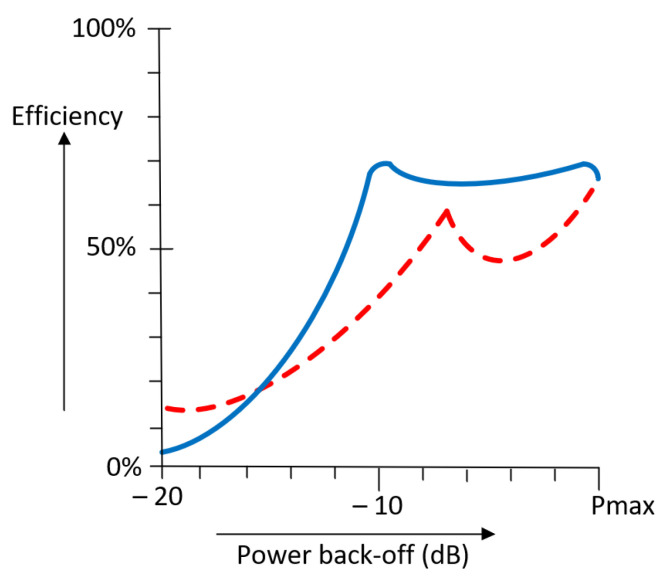
Outphasing efficiency (blue line) compared to Doherty amplifier (red line).

Figure 15 shows a comparison between the efficiencies of the Doherty and the outphasing topologies. Interestingly, the shape of the efficiency curve of the outphasing topology has two efficiency peaks like that of the Doherty. As in the Doherty PA, the outphasing efficiency changes depending on the desired OBO. Based on the previous analysis, it is evident that the outphasing architecture can provide a higher efficiency at back-off levels since it maintains higher values between the two peaks.

### 3.2. Design and Implementation

In this subsection, the design of the outphasing amplifier is described. To obtain a high PAPR, a Class-B amplifier was used. This configuration provides a good trade-off between efficiency and linearity [21]. A 100 nm GaN-on-Si transistor was used to design the amplifier with a 12 V of drain voltage and −1.75 V of gate voltage.

**Figure 16:**
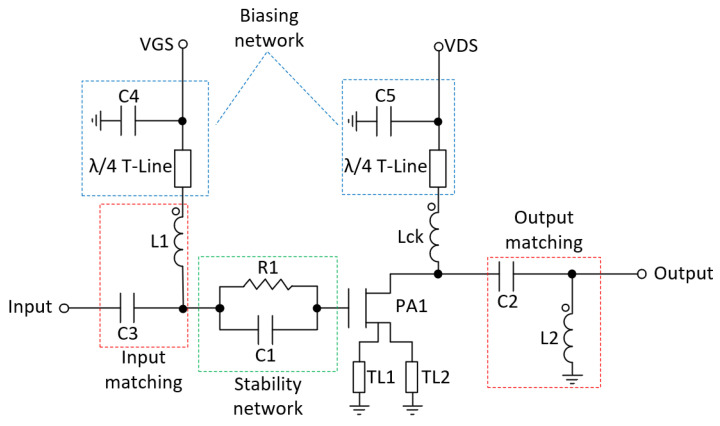
Schematic of a single branch outphasing amplifier.

To ensure low-frequency stability, a stabilization network on the drain and gate bias lines was added as seen in Figure 16 [25]. Additionally, a stability network composed by capacitor C1 and resistor R1 was added at the gate of the transistor to guarantee the stability of the RF power transistor [7,21]. Finally, inductors L2 and L3 and capacitors C2 and C3 were used to match the input and the output of the transistor.

**Figure 17:**
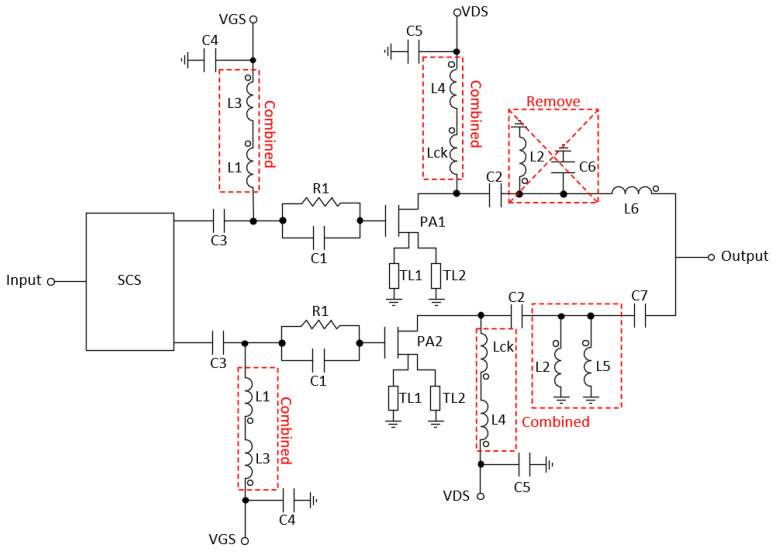
Outphasing amplifier schematic.

As in the case of the Doherty amplifier, due to large area of the TL, they were replaced by lumped-elements. Thus, the λ/4 T-line of the biasing network was replaced by inductors L4 and L3 as seen in Figure 17 [23].

The main function of the SCS is to translate the input AM signal into two PM signals with opposite phases. This component is complex to design, and both digital and analog implementations can be found in the literature [26,27]. The design of this component implies an increase in the complexity and power consumption of the circuit. For this reason, in this work, it has been considered that the SCS is an external element, and an ideal component has been used for the simulations.

The power combiner is the most critical part in the design of outphasing amplifiers. This component has two major functions. The first one is to convert the conceptual floating load into a single-ended load; the second one is to control the varying loading impedances “seen” by each line of the PA. Power combining is often implemented using variations of the outphasing approach. The transformer is closer to the theoretical development, the quarter-wavelength structure is more often used in hybrid RF PAs due to its easy microstrip structures fabrication and the asymmetric-transmission line has been more recently developed and enables the usage of efficient Class-E modes in the branch PAs [28]. In this case, the amplified AM signal at the output was initially combined using a λ/4 T-Line. However, due to the large area of this line, it was replaced by a lumped LC balun composed by inductors L6 and L7 and capacitors C6 and C7. The schematic of the outphasing PA design is shown in Figure 17.

**Figure 18:**
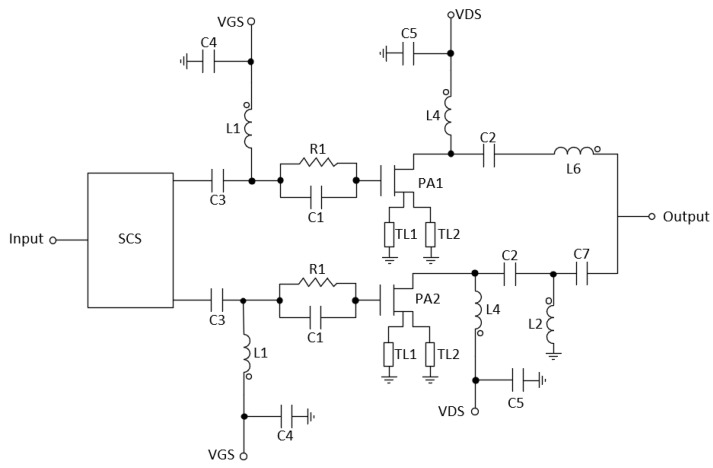
Final Outphasing amplifier schematic.

To simplify the number of components, and therefore the area of the circuit, some elements were combined or removed. Thus, L2 and C6 from PA1 were removed because they are in parallel and their equivalent admittance is almost null. Inductors L3 and L1 were combined, as well as L4 and Lck and also L2 and L5. The resulting scheme of the outphasing PA is shown in Figure 18. The component values are shown in Table 2.

**Figure 19:**
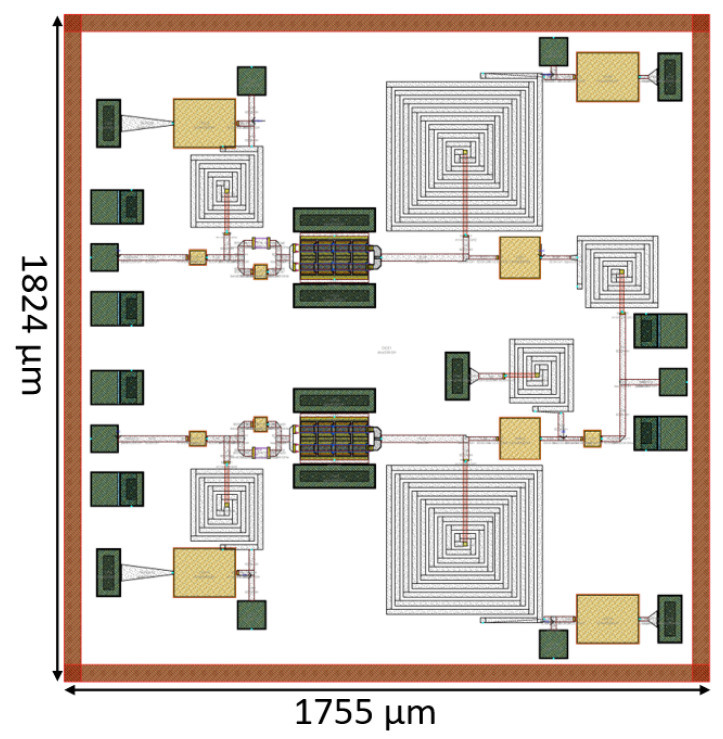
Outphasing layout.

Figure 19 displays the layout of the outphasing power amplifier (PA), which occupies an area of 1755 μm × 1824 μm, including the DC, input and output PADs.

## 4. Comparative Analysis

**Figure 20:**
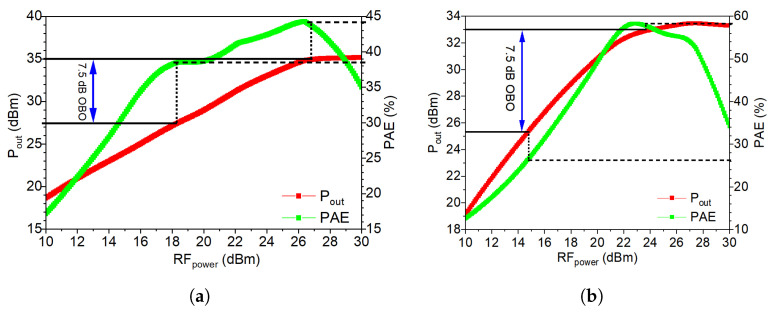

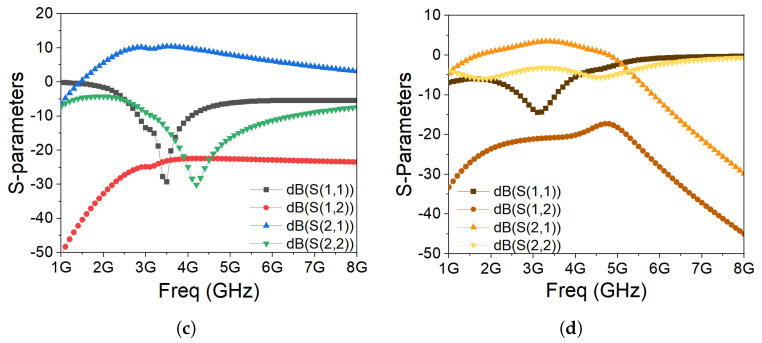
Post-layout simulation results at 3.6 GHz. (**a**) Large-signal Doherty PA. (**b**) Large-signal outphasing PA. (**c**) Small-signal outphasing PA. (**d**) Small-signal Doherty PA.

Figure 20a,b show the output power and PAE plotted as a function of the input power for the DPA and OPA, respectively. As can be seen in Figure 20a, the DPA provides a saturation power of 35 dBm and for that point, the PAE is 44.2%. For an OBO of 7.5 dB, the PAE of the DPA is 38.5%. On the other hand, Figure 20b shows that the saturation power of the OPA is 33 dBm and for that point, its efficiency is 58.3%, while at an OBO of 7.5 dB, the efficiency decreases to 26.1%. Figure 20c,d illustrate the small-signal post-layout simulation results for both PAs. At 3.6 GHz, the DPA demonstrates a gain of 9.4 dB, whereas the outphasing PA exhibits a gain of 10.4 dB. The outphasing PA shows superior input matching compared to the DPA, with an $S_{11}$ of −20.3 dB, contrasting with −8 dB for the DPA.

**Figure 21:**
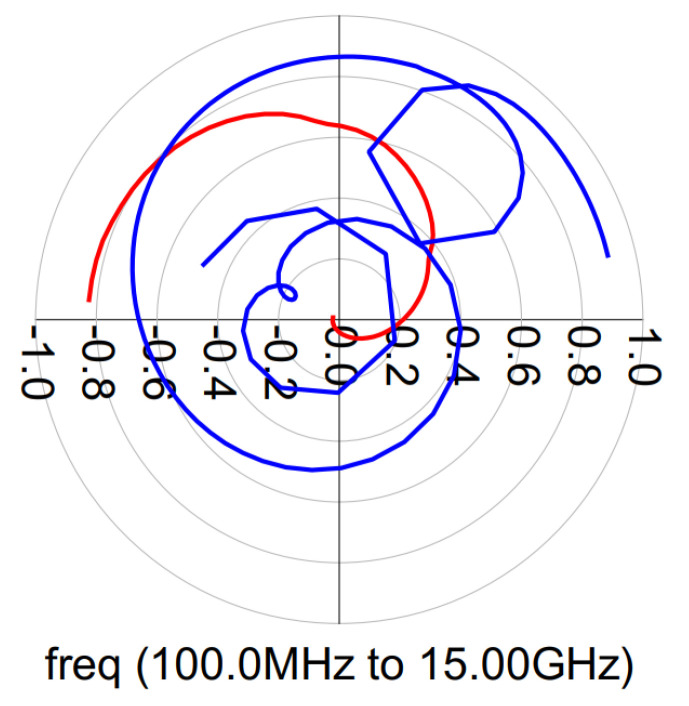
Outphasing even-mode stability analysis for the input (in red) and the output (in blue).

**Figure 22:**
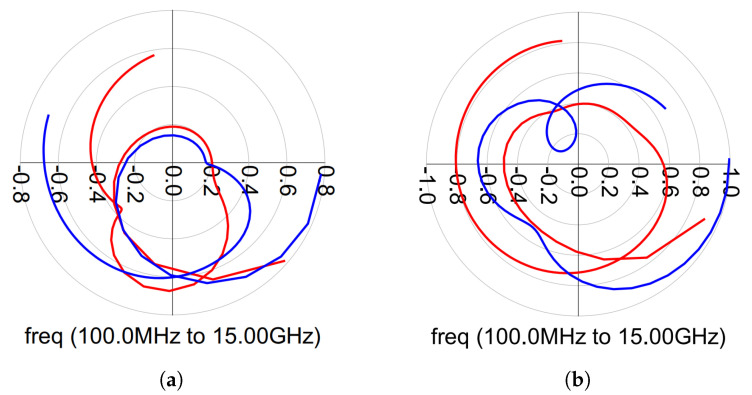
Doherty even-mode stability analysis for the input (in red) and the output (in blue). (**a**) Main amplifier. (**b**) Auxiliary amplifier.

The stability analysis can be divided into two groups: even-mode stability and odd-mode stability analyses. In the even-mode stability analyses, the Rollett stability factor (K) and the Nyquist stability criteria were calculated and, in the odd-mode stability, the Nyquist stability criterion was calculated. From the S-parameter simulation results, the K factor was greater than 1 (K > 1) [21]. However, checking the K-factor of the overall design was not enough, because Rollett’s K-factor guarantees the stability throughout the entire amplifier while the Nyquist stability criterion is usually used to ensure the stability of each individual stage. Hence, instabilities may appear at low frequencies. According to the Nyquist stability criterion, instabilities can occur if the magnitude is greater than 1 (>1) and encircle the (−1 + 0j) the point in a counter-clockwise direction. For the even-mode stability analysis, we employed the method described in [29]. Figure 21 and Figure 22 present the OPA and DPA even-mode stability simulation results. As observed, both amplifiers exhibit stable behavior.

**Figure 23:**
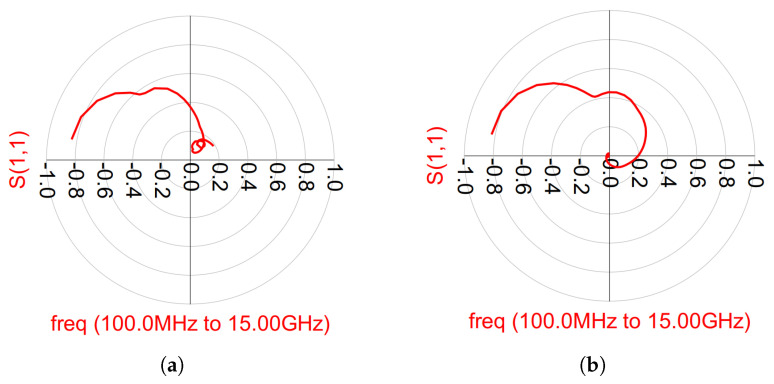
Outphasing odd-mode stability simulation results. (**a**) Amplifier 1. (**b**) Amplifier 2.

**Figure 24:**
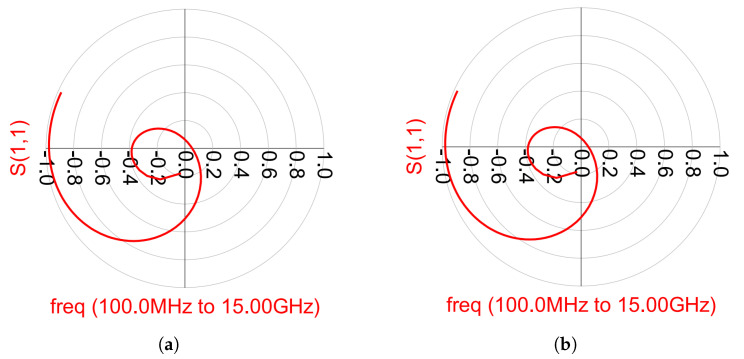
Doherty odd-mode stability simulation results. (**a**) Main amplifier. (**b**) Auxiliary amplifier.

On the other hand, odd-mode instability is more likely to occur in multi-device amplifiers due to different transistor characteristics and matching techniques. In Ref. [30], stability analysis is presented, examining the open-loop transfer functions at the junctions between passive and active devices. To ensure loop stability, none of these functions can encircle the critical point (1 + 0j) in a clockwise direction. Figure 23a,b display the simulation results for odd-mode stability of the OPA, while Figure 24a,b demonstrate the odd-mode stability simulation results of the main and auxiliary amplifiers in the DPA. Based on the plotted results of the odd-mode stability analysis, both amplifiers exhibit stable behavior.

**Figure 25:**
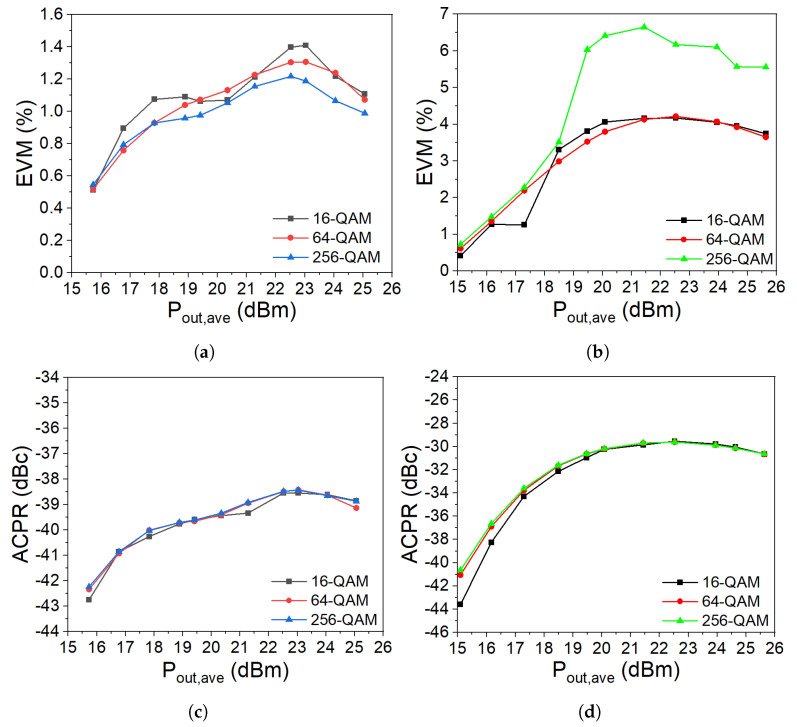
Simulated EVM and ACPR (average of lower and upper channels) versus the average output power for 16/64/256-QAM modulations with 100 MHz bandwidth. (**a**) Doherty EVM. (**b**) Outphasing EVM. (**c**) Doherty ACPR. (**d**) Outphasing ACPR.

To further evaluate the amplifier performance, both PAs were tested with an OFDM modulated signal with 7.5 dB PAPR. The simulations were carried out at 3.6 GHz without using any digital predistortion, and the signal bandwidth was 100 MHz. Figure 25 shows the simulated error vector magnitude (EVM) and the adjacent channel power ratio (ACPR) (average of lower and upper channels) as a function of average output power ($P_{out,ave}$) for different QAM signals. Figure 25a shows that for a low power level, the EVM was −40 dB (0.5%), and for a high-power level, the EVM reached up to −35 dB (1.05%) for the Doherty amplifier. Figure 25b shows that at low power, the EVM in the Outphasing amplifier was between −38 dB (0.7%) and −45 dB (0.4%), but for a higher power level, the EVM value increased significantly reaching up to −19 dB (6%) at some points for the 256-QAM signal. Figure 25c,d show the ACPR simulations of the Doherty and Outphasing amplifiers. As seen in the figures, the ACPR was not affected by the different modulations.

The performance of the designed Doherty and Outphasing PAs is summarized in Table 3 and compared to another reported sub-6 GHz GaN DPAs and OPAs. Regarding the DPA, it can be observed that only reference [31], achieves a higher PAE at a 1-dB compression point. However, it is important to mention that they use drain efficiency as a measure of PAE. In the case of the OPA, all the circuits except for [32] are designed with discrete components and use a preamplifier. Additionally, they also use the drain efficiency to measure the PAE; this is why they have a higher PAE value. As can be seen, our designs occupy an area of 3.26 mm^2^ for the DPA and 3.18 mm^2^ for the OPA, which are the smallest compared to the state-of-the-art, only followed by [33]. Therefore, the performance of the designed circuits is within the state of the art.

## 5. Conclusions

This paper presents a comparative study between Doherty and Outphasing PAs for 5G base stations. A theoretical analysis of both amplifiers has been conducted, and their designs have been carried out. The DPA outperforms the OPA in terms of output power, but at maximum output power, the DPA efficiency is lower than that of the OPA. Specifically, at the 1-dB compression point, the DPA achieves an output power of 35 dBm and a PAE of 44.2%, while the OPA achieves an output power of 33 dBm and a PAE of 58.3%. On the other hand, the DPA exhibits superior linearity and efficiency compared to the OPA at a 7.5-dB output back-off. Specifically, the ACPR for 64-QAM modulation and the PAE at 7.5 dB OBO for the DPA are −38.6 dBc and 38.5%, respectively, while for the OPA, these values are −30.6 dBc and 26.1%. The proposed designs have been compared with similar state-of-the-art designs, and in terms of area, they exhibit the smallest footprint. Due to its superior performance in back-off, it can be concluded that the DPA is the optimal choice for 5G base station applications.

## Figures and Tables

**Table 1:** Doherty component values.

Component	Value
Main PA	Wfg = 200 μm, Nfg = 4
Aux PA	Wfg = 200 μm, Nfg 6
TL1, TL2, TL3 & TL4	W = 200 μm, L = 10 μm
R1	34 Ω
C1	6 pF
C2	3.4 pF
L9	2.5 nH, Q = 8.7
C11	691 fF
C3	4.1 pF
C6 & C8	1.1 pF
L5 & L6	2.2 nH, Q = 8.4
L7 & L8	5.2 nH, Q = 9.3
R2	50 Ω
C5	8.7 pF
C4	1.4 pF
L4	4.3 nH, Q = 9.3

**Table 2:** Outphasing component values.

Component	Value
PA1 & PA2	Wfg = 200 μm, Nfg = 4
TL1 & TL2	W = 200 μm, L = 10 μm
R1	50 Ω
C1	600 fF
C2	2.5 pF
C3	750.9 fF
L1	2.1 nH, Q = 8.9
L4	14.95 nH, Q = 5
C4 & C5	12 pF
L2	9.2 nH, Q = 8.2
L6	2.2 nH, Q = 8.7
C7	884 fF

**Table 3:** Comparison of state-of-the-art sub-6 GHz Doherty and Outphasing PAs.

Ref.	Arch.	Tech. (nm)	*F_req._* (GHz)	*P_sat_* (dBm)	*PAE_1dB_* (%)	*OBO* (dB)	*PAE_OBO_* (%)	Gain_OBO_ (dB)	*Mod.*	*BW* (MHz)	*P_out,ave_* (dBm)	*EVM* (dB)	*ACPR* (dBc)	*Area* (mm^2^)
[8]	DPA	250	4.5–6	35–36	31.8–40.7	6	22.5–27.6	7.6–11.6	64-QAM	100	29.3	−30.5	−34.8/−37.5	8.4
[34]	DPA	250	3.6	38.8	33–55	8.5	40–46	11.7	N/A	N/A	N/A	N/A	N/A	8.58
[33]	DPA	250	5.1–5.9	36–38.7	43.2–47.3	6	31.6–49.5	14.4–17.3	256-QAM	80	25.7	<−30	N/A	3.88
[31]	DPA	250	3.5	36.8–37.2	58–70 ${}^{1}$	9	37–48 ${}^{1}$	10.2–12.5	64-QAM	20	26.9	N/A	−22.7/−23.0	9.75
**T.W.**	DPA	100	3.6	35	44.2	7.5	38.5	9.1	64-QAM	100	24.05	−34.4	−38.6/−38.6	3.26
[32]	OPA	100	2.6–3.6	>43	51.7	N/A	N/A	N/A	N/A	N/A	N/A	N/A	N/A	N/A
[35]	OPA	250	2.14	44.49	78 ${}^{1}$	8.5	60 ${}^{1}$	N/A	64-QAM	20	N/A	0.9 ${}^{2}$	−39.5	NA
[36]	OPA	100	3.5	43.5	64.1 ${}^{1}$	6	50.1 ${}^{1}$	N/A	WCDMA	5	N/A	N/A	−45	89.62
[37]	OPA	100	3.92	37	77 ${}^{1}$	7	>50 ${}^{1}$	N/A	N/A	N/A	N/A	N/A	N/A	NA
**T.W.**	OPA	100	3.6	33	58.3	7.5	26.1	10.6	64-QAM	100	25.6	−25.05	−30.6/−30.6	3.18

^1^ Drain efficiency; ^2^ EVM (%).

## References

[1] (2017). Itu-r. Minimum Requirements Related to Technical Performance for IMT-2020 Radio Interface(s) M Series Mobile, Radiodetermination, Amateur and Related Satellite Services. https://www.itu.int/pub/R-REP-M.2410-2017.

[2] (2018). Ee. TR 103 542—V1.1.1—Environmental Engineering (EE); Study on Methods and Metrics to Evaluate Energy Efficiency for Future 5G Systems. https://www.etsi.org/deliver/etsi_tr/103500_103599/103542/01.01.01_60/tr_103542v010101p.pdf.

[3] Parkvall S., Blankenship Y., Blasco R., Dahlman E., Fodor G., Grant S., Stare E., Stattin M. (2020). 5G NR Release 16: Start of the 5G Evolution. IEEE Commun. Stand. Mag..

[4] Thantharate A., Beard C., Marupaduga S. An Approach to Optimize Device Power Performance Towards Energy Efficient Next Generation 5G Networks. Proceedings of the 2019 IEEE 10th Annual Ubiquitous Computing, Electronics and Mobile Communication Conference, UEMCON 2019.

[5] (2019). TSGR. TS 138 104—V15.5.0—5G; NR; Base Station (BS) Radio Transmission and Reception (3GPP TS 38.104 Version 15.5.0 Release 15). https://www.etsi.org/deliver/etsi_ts/138100_138199/138104/15.05.00_60/ts_138104v150500p.pdf.

[6] Shurdi O., Ruci L., Biberaj A., Mesi G. (2021). 5G Energy Efficiency Overview. Eur. Sci. J. ESJ.

[7] Cripps S.C. (2006). RF Power Amplifiers for Wireless Communications.

[8] Nikandish G., Staszewski R.B., Zhu A. (2019). Bandwidth enhancement of GaN MMIC Doherty power amplifiers using broadband transformer-based load modulation network. IEEE Access.

[9] Ning K., Fang Y., Hosseinzadeh N., Buckwalter J.F. (2020). A 30-GHz CMOS SOI Outphasing Power Amplifier with Current Mode Combining for High Backoff Efficiency and Constant Envelope Operation. IEEE J. Solid-State Circuits.

[10] Garcia J.A., Ruiz M.N., Cordero A., Vegas D. (2021). Current-Injected Load-Modulated Outphasing Amplifier for Extended Power Range Operation. IEEE Microw. Wirel. Compon. Lett..

[11] Lazarevic V.Z., Vasic M., Garcia O., Alou P., Oliver J.A., Cobos J.A. (2020). System Linearity-Based Characterization of High-Frequency Multilevel DC-DC Converters for S-Band EER Transmitters. IEEE J. Emerg. Sel. Top. Power Electron..

[12] Wang F., Yang A.H., Kimball D.F., Larson L.E., Asbeck P.M. (2005). Design of wide-bandwidth envelope-tracking power amplifiers for OFDM applications. IEEE Trans. Microw. Theory Tech..

[13] Wang Z. (2014). Envelope Tracking Power Amplifiers for Wireless Communications.

[14] Kahn L.R. (1952). Single-Sideband Transmission by Envelope Elimination and Restoration. Proc. IRE.

[15] Su D.K., McFarland W.J. (1998). An IC for linearizing RF power amplifiers using envelope elimination and restoration. IEEE J. Solid-State Circuits.

[16] Doherty W.H. (1936). Technical Papers: A New High Efficiency Power Amplifier for Modulated Waves. Proc. Inst. Radio Eng..

[17] Chireix H. (1935). High power outphasing modulation. Proc. Inst. Radio Eng..

[18] Wang W., Chen S., Cai J., Zhou X., Chan W.S., Wang G. (2020). A compact outphasing power amplifier with integrated reactive compensation. Microw. Opt. Technol. Lett..

[19] Kim B. (2018). Doherty Power Amplifiers: From Fundamentals to Advanced Design Methods.

[20] Chen S., Xue Q. (2012). Optimized load modulation network for doherty power amplifier performance enhancement. IEEE Trans. Microw. Theory Tech..

[21] Bahl I.J. (2008). Fundamentals of RF and Microwave Transistor Amplifiers.

[22] Elmala M., Bishop R. A 90 nm CMOS Doherty power amplifier with integrated hybrid coupler and impedance transformer. Proceedings of the Digest of Papers—IEEE Radio Frequency Integrated Circuits Symposium.

[23] Vogel R.W. (1992). Analysis and Design of Lumped- and Lumped-Distributed-Element Directional Couplers for MIC and MMIC Applications. IEEE Trans. Microw. Theory Tech..

[24] Li S., Huang M.Y., Jung D., Huang T.Y., Wang H. (2021). A MM-Wave Current-Mode Inverse Outphasing Transmitter Front-End: A Circuit Duality of Conventional Voltage-Mode Outphasing. IEEE J. Solid-State Circuits.

[25] Florian C., Traverso P.A., Santarelli A., Filicori F. (2013). An active bias network for the characterization of low-frequency dispersion in high-power microwave electron devices. IEEE Trans. Instrum. Meas..

[26] Martin D.N., Barton T.W. (2021). Inphasing Signal Component Separation for an X-Band Outphasing Power Amplifier. IEEE Trans. Microw. Theory Tech..

[27] Panseri L., Romanò L., Levantino S., Samori C., Lacaita A.L. Low-power all-analog component separator for an 802.11a/g LINC transmitter. Proceedings of the ESSCIRC 2006—Proceedings of the 32nd European Solid-State Circuits Conference.

[28] Litchfield M., Cappello T. (2019). The Various Angles of Outphasing PAs: Competitiveness of Outphasing in Efficient Linear PA Applications. IEEE Microw. Mag..

[29] Değirmenci A. (2014). A KU-Band Phemt Mmic High Power Amplifier Design. https://core.ac.uk/download/pdf/52928744.pdf.

[30] Ohtomo M. (1993). Stability Analysis and Numerical Simulation of Multidevice Amplifiers. IEEE Trans. Microw. Theory Tech..

[31] Zhao Y., Li X., Gai C., Liu C., Qi T., Hu B., Hu X., Chen W., Helaoui M., Ghannouchi F.M. (2021). Theory and design methodology for reverse-modulated dual-branch power amplifiers applied to a 4G/5G broadband GaN MMIC PA design. IEEE Trans. Microw. Theory Tech..

[32] Zhu Y., Cheng Z., Chen Y., Liu G. Design of a broadband chireix combiner based on class-f power amplifier. Proceedings of the 2019 8th International Symposium on Next Generation Electronics, ISNE 2019.

[33] Li S.H., Hsu S.S., Zhang J., Huang K.C. (2018). Design of a Compact GaN MMIC Doherty Power Amplifier and System Level Analysis With X-Parameters for 5G Communications. IEEE Trans. Microw. Theory Tech..

[34] Seidel A., Wagner J., Ellinger F. 3.6 GHz asymmetric Doherty PA MMIC in 250 nm GaN for 5G applications. Proceedings of the 2020 German Microwave Conference (GeMiC).

[35] Afanasyev P., Grebennikov A., Farrell R., Dooley J. (2020). Analysis and Design of Outphasing Transmitter Using Class-E Power Amplifiers with Shunt Capacitances and Shunt Filters. IEEE Access.

[36] Wang W., Chen S., Cai J., Zhou X., Chan W.S., Wang G. (2021). A high efficiency dual-band outphasing power amplifier design. Int. J. RF Microw. Comput.-Aided Eng..

[37] Ogasawara R., Takayama Y., Ishikawa R., Honjo K. (2020). A 3.9-GHz-Band outphasing power amplifier with compact combiner based on dual-power-level design for wide-dynamic-range operation. IEEE MTT-S Int. Microw. Symp. Dig..

